# Glycemic control and its influencing factors in type 2 diabetes patients in Anhui, China

**DOI:** 10.3389/fpubh.2022.980966

**Published:** 2022-10-04

**Authors:** Xiu-Ya Xing, Xin-Yi Wang, Xi Fang, Jing-Qiao Xu, Ye-Ji Chen, Wei Xu, Hua-Dong Wang, Zhi-Rong Liu, Sha-Sha Tao

**Affiliations:** ^1^Department of Chronic Non-communicable Disease Prevention and Control, Anhui Provincial Center for Disease Control and Prevention, Hefei, China; ^2^Department of Radiation Oncology, The First Affiliated Hospital of Anhui Medical University, Hefei, China; ^3^First Clinical Medical College, Anhui Medical University, Hefei, China; ^4^Department of Epidemiology and Biostatistics, School of Public Health, Anhui Medical University, Hefei, China

**Keywords:** type 2 diabetes (T2D), glycemic control, epidemiological characteristics, influencing factors, health education and promotion

## Abstract

**Objective:**

To investigate the status of glycemic control and analyze its influencing factors in patients with type 2 diabetes (T2D) in Anhui, China.

**Methods:**

1,715 T2D patients aged 18–75 years old were selected from 4 counties or districts in Anhui Province in 2018, using a convenience sampling method. All patients have undergone a questionnaire survey, physical examination, and a glycosylated hemoglobin (HbA1c) test. According to the 2022 American Diabetes Association criteria, HbA1c was used to evaluate the glycemic control status of patients, and HbA1c < 7.0% was defined as good glycemic control. The influencing factors of glycemic control were analyzed by multivariate unconditional logistic regression.

**Results:**

The prevalence of good glycemic control among people with T2D in the Anhui Province was low (22.97%). On univariate analysis, gender, education level, occupation, region, smoking, drinking, waist circumference and disease duration (all *P* < 0.05) were significantly associated with glycemic control. The factors associated with pool glycemic control were female gender [OR = 0.67, 95%CI (0.52, 0.86), *P* = 0.001], higher level of education [OR = 0.47, 95%CI (0.27, 0.83), *P* = 0.001], living in rural areas [OR = 1.77, 95%CI (1.39, 2.26), *P* < 0.001], central obesity [OR = 1.58, 95%CI (1.19, 2.09), *P* = 0.001] and longer duration of disease [OR = 2.66, 95%CI (1.91, 3.69), *P* < 0.001].

**Conclusions:**

The prevalence of good glycemic control in people with T2D in Anhui Province was relatively low, and gender, region, education level, central obesity and course of the disease were influencing factors. The publicity and education on the importance of glycemic control should be further strengthened in T2D patients, and targeted intervention measures should be carried out for risk groups.

## Introduction

Diabetes has become an important public health problem in China. Currently, there are an estimated 114 million people with diabetes in China (1). According to statistics in 2013, about a quarter of diabetes-related deaths worldwide occurred in China (2). China has experienced one of the largest increases in the prevalence of diabetes globally and the significant increase in the prevalence of diabetes is largely attributable to type 2 diabetes (T2D) (3). It is well known that diabetes is a polygenic genetic metabolic disease that is susceptible to multiple environmental factors and affects the entire body (4). In the past two decades, China's economy has developed rapidly and lifestyles have undergone major changes. These lifestyle factors, such as lack of exercise, unhealthy diet, and obesity are all related to the etiology of T2D (5). T2D can lead to multiple complications, including retinopathy, kidney disease, neuropathy, skin ulcers, and atherosclerosis, which can reduce the quality of life, result in premature death, and cause a great economic burden to individuals and society (6). However, the current status of treatment and control of T2D in China is not optimistic (7).

Adequate glycemic control is of great significance for delaying the development of T2D and reducing the complications of T2D (8). Inadequate glycemic control may lead to symptoms of hyperglycemia (polyuria, polydipsia) and its direct complications (poor wound healing, dehydration, hypertonic hyperglycemia syndrome, diabetic ketoacidosis, diabetic coma) (9). Long-term and moderate glycemic control can reduce the incidence of microvascular and macrovascular complications (10). A recent study showed that only half of patients receiving diabetes treatment had sufficient glycemic control (11), and insufficient glycemic control was increasingly common in patients with diabetes. Excessive body fat accumulation is closely related to poor glycemic control brought on by impaired insulin signaling, Gummesson A et al. demonstrated weight loss promoted glycemic control improvement (12). The effects of age and sex on glycemic control remain controversial and other influencing factors are financial hardship, psychosocial, and education level et al. (13, 14). Due to differences in economic level, population size, environment and dietary habits among provinces and regions in China, the prevalence and control status of T2D also have complex geographical distribution (1).

Among these provinces, Anhui Province has caught our attention. The prevalence of total diabetes in Anhui was 8.5% and the prevalence in central Anhui was 10.17% which was comparable to China (11.2%) (15, 16). Overall, the prevalence of diabetes in Anhui is high, many diet and lifestyle habits of residents in Anhui Province are closely related to the possible influencing factors of glycemic control. Furthermore, the population base of Anhui Province is large, so we speculate that the T2D glycemic control states of Anhui Province is likely to be worse and worse because of these factors. However, until now, the relationship between the T2D glycemic control and these factors in Anhui Province is still unclear.

Based on the community population, this study conducted a research on T2D patients among residents in Anhui Province, investigated the glycemic control status of T2D patients and its influencing factors, so as to provide a theoretical basis for later proposed targeted interventions to improve the glycemic control of T2D patients and reduce the incidence of complications.

## Objects and methods

### Study design and participants

The study was a cross-sectional study that designed to assess the status of glycemic control among T2D patients in Anhui Province and explore the related influencing factors. A total of 1,715 T2D patients took part in this trial. Ethics committee of the Anhui Medical University reviewed and approved the study, written informed consents to participate in this study have been provided by participants.

### Sampling

The investigation was carried out from August to December 2018. A convenience sampling approach with a four-level quality control was adopted (17). Inclusion criteria included: (1) aged 18–75; (2) normal cognitive ability; (3) diagnosed with T2D at the hospital. Four counties or districts were selected from the national surveillance sites of chronic diseases and risk factors in Anhui Province. Finally, 1,715 T2D patients were included in this survey. 18–44 years old accounted for 16%, 45–59 years old accounted for 44%, 60–75 years old accounted for 40%.

### Methods

#### Questionnaire-sociodemographic data and lifestyle behaviors

All participants were subjected to questionnaire surveys in order to gather sociodemographic data and lifestyle behaviors. Investigators conducted the questionnaire survey in the form of face-to-face interviews using tablet computers after receiving a unified training course. The questionnaire contained basic information such as gender, age, profession and so on, as well as lifestyle information such as smoking, drinking, exercise intervention, dietary control, duration and family history of T2D.

#### Physical examination-anthropometric measurements

Physical examination was to measure height, weight, waist circumference, etc. TGZ type height and sitting height meter with a maximum scale of 2.0 m and an accuracy of 0.1 cm was used for height measurement; TANITA HD-390 type scale with a minimum unit of 0.1 kg was used for weight measurement; a torch-type waist ruler with a maximum scale of 1.5 m, a width of 1 cm and an accuracy of 0.1 cm was used for waist circumference measurement. Body mass index (BMI) was used to define overweight and obesity, of which BMI <18.5 was defined as underweight, 18.5 ≤ BMI < 24.0 was defined as normal weight, 24.0 ≤ BMI < 28.0 was defined as overweight, and BMI≥28.0 was defined as obesity (18). Central obesity was defined according to waist circumference, where waist circumference <85 cm in men and <80 cm in women were defined as normal waist circumference; 85 ≤ waist circumference < 90 in men and 80 ≤ waist circumference <85 in women were defined as pre-central obesity; and waist circumference ≥90 cm in men and ≥85 cm in women were defined as central obesity (17).

#### HbA1C-long term glycemic control

Venous blood was collected from all survey subjects after fasting for 10–12 h to detect glycosylated hemoglobin (HbA1c) which was used to evaluate the states of glycemic control. According to the 2022 American Diabetes Association criteria, HbA1c < 7.0% was defined as good glycemic control (19).

### Quality control

Strict quality control links were set up in the early stage of the survey, during the survey and after the survey to ensure the reliability of the data. The provincial project team organized unified training and went to all survey sites for on-site guidance and quality control. Each investigation site used uniform survey tools and questionnaires, as well as a dedicated quality controller in charge of daily quality control. Epidata 3.1 double entry form was used for data entry.

### Statistical analysis

SPSS 20.0 was used for data sorting and statistical analysis. Sociodemographic characteristics were described using frequencies for categorical variables, mean and standard deviation (SD) for continuous variables. The χ^2^ test was used to compare the glycemic control states of T2D patients with sociodemographic and lifestyle factors. To identify the variables that affect glycemic control in T2D patients, a univariate analysis was performed first. The variables that were statistically significant (*P*<0.05) were then adapted to multivariate unconditional logistic regression analysis. The significance tests were two-sided, with a *P-*value ≤ 0.05 considered statistically significant.

## Results

### Sociodemographic characteristics of T2D patients

A total of 1,715 T2D patients were included in this survey. 394 T2D patients (22.97%) had good glycemic control in Anhui Province. Among them, males were lower than females (χ^2^ = 6.59, *P* = 0.010); primary school was the lowest, and college education was the highest (χ^2^ = 14.31, *P* = 0.003); rural was lower than urban (χ^2^ = 24.15, *P* < 0.001); different professions had differences (χ^2^ = 11.25, *P* = 0.024). The demographic characteristics of all participants are shown in Table 1.

**Table 1:** The glycemic control of T2D patients with sociodemographic characteristics.

**Characteristic**	**Good glycemic control** ***n* %**	**Uncontrolled blood sugar** ***n* %**	**χ^2^ value**	***P-*value**
Gender			6.59	0.010*
Male	172 (20.33)	674 (79.67)		
Female	222 (25.55)	647 (74.45)		
Age (years old)			4.59	0.101
18~	55 (25.94)	157 (74.06)		
45~	158 (20.60)	609 (79.40)		
60~75	181 (24.59)	555 (75.41)		
Educational level			14.31	0.003*
Illiterate/semi-literate	239 (23.05)	798 (76.95)		
Primary school	88 (19.34)	367 (80.66)		
High school/secondary school/technical school	42 (26.25)	118 (73.75)		
College and above	25 (39.68)	38 (60.32)		
Profession			11.25	0.024*
Agriculture, forestry, animal husbandry and fisheries	113 (19.96)	453 (80.04)		
Production transportation/commercial services	41 (18.30)	183 (81.70)		
Institutions, institutions and professional and technical personnel	34 (26.77)	93 (73.23)		
retired	58 (23.67)	187 (76.33)		
Housework/unemployed, etc. others	148 (26.76)	405 (73.24)		
Marital status			2.91	0.233
Not married	9 (37.50)	15 (62.50)		
Married/Cohabiting	358 (22.74)	1,216 (77.26)		
Divorced/separated/widowed	27 (23.08)	90 (76.92)		
Annual household income (yuan)			2.86	0.414
<20,000	91 (26.00)	259 (74.00)		
20,000~	99 (23.46)	323 (76.54)		
≥50,000	123 (21.43)	451 (78.57)		
Not sure/refused to answer	81 (21.95)	288 (78.05)		
Region			24.15	<0.001*
Urban	233 (28.14)	595 (71.86)		
Rural	161 (18.15)	726 (81.85)		
Total (*n =* 1,715)	394 (22.97)	1,321 (77.03)		

^*^P-value < 0.05.

### Lifestyle factors of T2D patients

The status of glycemic control were different in T2D patients with different lifestyle factors. The research finding has shown that the good glycemic control prevalence of current smokers was lower than non-smokers (χ^2^ = 4.69, *P* = 0.030), and they had drunk alcohol in the past 30 days lower than those who had never drunk alcohol (χ^2^ = 6.07, *P* = 0.014); the larger the waist circumference, the lower the blood glycemic control prevalence (χ^2^ = 10.15, *P* = 0.006); with the increase of duration of disease, the blood glycemic control prevalence showed a downward trend (χ^2^ = 35.04, *P* < 0.001). The glycemic control in T2D patients with different lifestyle factors is shown in Table 2.

**Table 2:** The glycemic control of T2D patients with lifestyle factors.

**Characteristic**	**Good glycemic control** ***n* %**	**Uncontrolled blood sugar** ***n* %**	**χ^2^ value**	***P-*value**
Current smoker			4.69	0.030*
Yes	74 (18.93)	317 (81.07)		
Not	320 (24.17)	1,004 (75.83)		
Drinking alcohol in the past 30 days			6.07	0.014*
Yes	91 (18.96)	389 (81.04)		
No	303 (24.53)	932 (75.47)		
BMI			3.75	0.154
<24	126 (26.03)	358 (73.97)		
24~	169 (21.39)	621 (78.61)		
28~	99 (22.45)	342 (77.55)		
Waistline			10.15	0.006*
Normal	109 (28.91)	268 (71.09)		
Central obesity prophase	74 (22.77)	251 (77.23)		
Central obesity	211 (20.83)	802 (79.17)		
Exercise intervention			0.17	0.682
Yes	177 (23.44)	578 (76.56)		
No	217 (22.60)	743 (77.40)		
Diet control			0.26	0.608
Yes	317 (22.72)	1,078 (77.28)		
No	77 (24.06)	243 (75.94)		
Participate in primary follow-up management			1.18	0.277
Yes	327 (22.51)	112,6 (77.49)		
No	67 (25.57)	195 (74.43)		
Duration of disease (years)			35.04	<0.001*
<3	117 (31.88)	250 (68.12)		
3~	127 (26.24)	357 (73.76)		
6~	63 (18.31)	281 (81.69)		
10~	87 (16.73)	433 (83.27)		
Have a family history			3.68	0.055
Yes	153 (20.73)	585 (79.27)		
No	241 (24.67)	736 (75.33)		
Total (*n =* 1,715)	394 (22.97)	1,321 (77.03)		

^*^P-value < 0.05.

### Univariate analysis of risk factors for glycemic control in T2D patients in Anhui Province

Sociodemographic characteristics and lifestyle factors were analyzed by univariate analysis. Analysis result showed that female [OR = 0.66, 95%CI (0.49, 0.90), *P* = 0.008], college education or above [OR = 0.38, 95%CI (0.20, 0.73), *P* = 0.004] were positively associated with glycemic control and rural region [OR = 1.84, 95%CI (1.39, 2.43), *P* = 0.004], central obesity [OR = 1.80, 95%CI (1.21, 2.68), *P* = 0.004], long duration of disease [OR = 2.76, 95%CI (1.95, 3.91), *P* < 0.001] were inversely correlated with glycemic control. The result of univariate analysis is shown in Table 3.

**Table 3:** Univariate analysis of glycemic control in patients with T2D.

**Characteristic**	***B-*value**	**Wald value**	**OR (95%CI)**	***P-*value**
Gender				
Male			1.00	
Female	−0.412	6.938	0.66 (0.49, 0.90)	0.008*
Age (years old)				
18~		4.162	1.00	0.125
45~	0.105	0.293	1.11 (0.76, 1.63)	0.588
60~75	−0.183	0.762	0.83 (0.55, 1.26)	0.383
Educational level				
Illiterate/semi-literate		10.681	1.00	0.014*
Primary school	0.057	0.123	1.06 (0.77, 1.45)	0.725
High school/secondary school/technical school	−0.321	1.928	0.73 (0.46, 1.14)	0.165
College and above	−0.974	8.358	0.38 (0.20, 0.73)	0.004
Profession				
Agriculture, forestry, animal husbandry and fisheries		3.570	1.00	0.467
Production transportation/commercial services	0.027	0.014	1.03 (0.66, 1.60)	0.907
Institutions, institutions and professional and technical personnel	−0.189	0.435	0.83 (0.47, 1.45)	0.510
retired	0.094	0.140	1.10 (0.67, 1.80)	0.709
Housework/unemployed, etc. others	−0.203	1.598	0.82 (0.60, 1.12)	0.206
Marital status				
Not married		3.869	1.00	
Married/cohabiting	0.829	3.303	2.29 (0.94, 5.60)	0.069
Divorced/separated/widowed	0.996	3.824	2.71 (1.00, 7.34)	0.051
Region				
Urban			1.00	
Rural	0.610	18.547	1.84 (1.39, 2.43)	<0.001*
Current smoker				
Yes			1.00	
Not	−0.018	0.010	0.98 (0.69, 1.40)	0.920
Drinking alcohol in the past 30 days				
Yes			1.00	0.055
No	−0.266	2.774	0.77 (0.56, 1.05)	0.096
Annual household income (yuan)				
<20,000		6.016	1.00	0.111
20,000~	0.188	1.029	1.21 (0.84, 1.73)	0.311
≥50,000	0.393	4.013	1.48 (1.01, 2.18)	0.045
Not sure/refused to answer	0.385	4.335	1.47 (1.02, 2.11)	0.037
BMI				
<24		1.040	1.00	0.595
24~	−0.124	0.489	0.88 (0.62, 1.25)	0.484
28~	−0.229	1.039	0.80 (0.51, 1.24)	0.308
Waistline				
Normal		8.467	1.00	0.014*
Central obesity prophase	0.297	2.304	1.35 (0.92, 1.98)	0.129
Central obesity	0.587	8.427	1.80 (1.21, 2.68)	0.004
Exercise intervention				
Yes			1.00	
No	0.029	0.048	1.03 (0.79, 1.34)	0.826
Diet control				
Yes			1.00	
No	0.019	0.014	1.02 (0.74, 1.40)	0.906
Participate in primary follow-up management				
Yes			1.00	
No	−0.271	2.586	0.76 (0.55, 1.06)	0.108
Duration of disease (years)	−0.018			
<3		40.127	1.00	<0.001*
3~	0.308	3.720	1.36 (1.00, 1.86)	0.054
6~	0.829	19.297	2.29 (1.58, 3.32)	<0.001
10~	1.016	32.973	2.76 (1.95, 3.91)	<0.001
Have a family history				
Yes			1.00	
No	−0.146	1.336	0.86 (0.67, 1.11)	0.248

^*^P-value < 0.05.

### Analysis of risk factors for glycemic control in T2D patients in Anhui Province

Gender, education level, occupation, region, smoking, drinking, waist circumference, disease duration, etc., were used as independent variables, and glycemic control was used as the dependent variable, and then multivariate analysis was performed using a multivariate logistic regression model. The results showed that female [OR = 0.67, 95%CI (0.52, 0.86), *P* = 0.001] and higher education level [OR = 0.47, 95%CI (0.27, 0.83), *P* = 0.001] were a protective factor for glycemic control in T2D patients, while rural area, central obesity, and long period of illness were risk factors (all *P* < 0.05). The results of multivariate logistic analysis are shown in Table 4.

**Table 4:** Multivariate unconditional logistic regression analysis of glycemic control in patients with T2D.

**Characteristic**	***B* value**	**Wald value**	**OR (95%CI)**	***P*-value**
Gender
Male			1.00	
Female	−0.404	10.240	0.67 (0.52, 0.86)	0.001*
Educational level
Illiterate/semi-literate			1.00	
Primary school	0.215	2.050	1.24 (0.92, 1.67)	0.152
High school/secondary school/technical school	−0.115	0.301	0.89 (0.60, 1.34)	0.583
College and above	−0.754	6.836	0.47 (0.27, 0.83)	0.009*
Region
Urban			1.00	
Rural	0.571	20.819	1.77 (1.39, 2.26)	<0.001*
Waistline
Normal			1.00	
Central obesity prophase	0.267	2.205	1.31 (0.92, 1.86)	0.138
Central obesity	0.457	10.239	1.58 (1.19, 2.09)	0.001*
Duration of disease (years)
<3			1.00	
3~	0.288	3.412	1.33 (0.98, 1.81)	0.065
6~	0.794	18.828	2.21 (1.55, 3.17)	<0.001*
10~	0.977	33.986	2.66 (1.91, 3.69)	<0.001*

^*^P-value < 0.05.

## Discussion

As a common chronic disease with increasing prevalence, the prevention and treatment of T2D and its complication are crucial (20); the treatment is glycemic control. Nevertheless, different geographical environment, socioeconomic conditions, dietary habits and health services cause varying level of glycemic control. In this study, we investigated the glycemic control status and their influencing factors in Anhui province.

This study showed that the prevalence of good glycemic control among T2D patients in Anhui Province was 22.97%, which was lower than the previously reported 39.70% (21), but significantly higher than 2.00% reported in Beijing (22). It is worth noting that in this survey, the good glycemic control prevalence of men was lower than that of women. The research result of Esteghamati A et al. was consistent with our findings (23). Nevertheless, in one study, female had the strong association for persistent poor glycemic control (24). The reason for the contradictory results could be due to different tobacco and alcohol consumption. According to previous study, when compared with non-smokers, both current and former smokers had poorer glycemic control, and there was a significant association between current smoking and deteriorating glycemic control (25). The study also found that smokers had lower glycemic control prevalence than non-smokers, and drinkers had worse glycemic control than non-drinkers. In addition, one study (26) conducted in the United States found that alcohol consumption was inversely related to glycemic control in diabetic patients and a correlation study found a dose-response relationship between active smoking and the risk of poor glycemic control in men (27).

The study also found that the urban population had a higher prevalence of good glycemic control than the rural population. The higher the education level, the higher good glycemic control prevalence. These results in accordance with the result of Nigussie S et al. (28) and Sonmez A et al. (29). Awareness of diabetes and socioeconomic status may have contributed to this outcome. Relevant studies have shown that people with higher education level and more knowledge about diabetes have better glycemic control (30, 31). Studies have reported that follow-up care for T2D was essential for improving population health in primary care (32). However, due to the low level of education and medical services in rural areas, the prevalence of T2D is rising rapidly in rural areas (33). Therefore, the government should strengthen primary health care services and implement educational interventions for T2D patients in rural areas (34, 35) to enable better disease management and glycemic control for rural patients.

Furthermore, the result was found that T2D patients with central obesity had poor glycemic control, suggesting that central obesity may be a risk factor or an outcome of poor glycemic control in T2D patients. It has been proposed that with the rapid increase in global obesity rates, the prevalence of T2D was also increasing (36). Wang Z et al. (37) also reported that central obesity might increase the risk of poor glycemic control. This might attribute to that central obesity is closely related to insulin resistance (38). Excessive energy intake will increase circulating glucose and free fatty acids, promote fat Oxidative stress in cells, skeletal muscle, pancreatic beta cells and hepatocytes, which alters insulin receptor signaling, reduces cellular utilization of glucose and the limited ability of adipocytes to store excess plasma-free fatty acids. They subsequently lead to fat accumulation in ectopic deposition in liver, skeletal muscle or cardiac muscle, resulting in insulin resistance in these tissues (39). For T2D patients with central obesity, intermittent eating can be utilized to manage blood glycemic levels, which has been shown to be effective (40).

In addition, this study also found that the good glycemic control prevalence was negatively related to the duration of disease. This is consistent with previous findings, which found that glycemic control worsened over the course of the disease in patients with T2D (41, 42). Early glycemic control has been shown to reduce the risk of complications and death in T2D patients, particularly in patients with a longer course of disease; the earlier glycemic control is initiated, the lower the risk of death (42). Previous studies have indicated that in T2D patients, measures such as enhanced education, nursing awareness, and increased diabetic self-testing can improve their glycemic control (43). Therefore, in response to this result, we should pay special attention to individuals with long-duration T2D patients, formulate long-term strategies and strengthen the publicity and education of patients regarding self-test diabetes in order to improve the states of glycemic control.

## Limitation

Our study has some limitations. Firstly, this study is a cross-sectional study, which can only find and analyze the influencing factors of glycemic control, but cannot infer the causal relationship between them. Secondly, the use of convenience sampling limited the representativeness and generalizability of the research for Anhui province. Lastly, not all possible influencing factors were included, such as health literacy, medication adherence et al., were not examined in this study.

## Conclusion

The prevalence of good glycemic control in T2D patients in Anhui Province was at a low level. The influencing factors of glycemic control were gender, region, education level, central obesity and course of the disease. We should further strengthen publicity and education on the importance of glycemic control to health in T2D patients, especially those lived in rural areas, with low education levels or with central obesity, in early disease. At the same time, the awareness of glycemic control and self-management should be improved in T2D patients.

## Data availability statement

The raw data supporting the conclusions of this article will be made available by the authors, without undue reservation.

## Ethics statement

The studies involving human participants were reviewed and approved by the Ethics Committee of the Anhui Medical University. The patients/participants provided their written informed consent to participate in this study.

## Author contributions

S-ST and Z-RL guided on the design and statistical analyses. X-YX, X-YW, and XF wrote the manuscript. X-YX had primary responsibility for final content. J-QX, Y-JC, WX, and H-DW contributed to data collection and interpretation of findings. All authors contributed to the manuscript editing, read, and approved the final manuscript.

## Conflict of interest

The authors declare that the research was conducted in the absence of any commercial or financial relationships that could be construed as a potential conflict of interest.

## Publisher's note

All claims expressed in this article are solely those of the authors and do not necessarily represent those of their affiliated organizations, or those of the publisher, the editors and the reviewers. Any product that may be evaluated in this article, or claim that may be made by its manufacturer, is not guaranteed or endorsed by the publisher.
